# The Precipitous Decline in Reasoning and Other Key Abilities with Age and Its Implications for Federal Judges

**DOI:** 10.3390/jintelligence9040052

**Published:** 2021-10-25

**Authors:** Alan S. Kaufman

**Affiliations:** Yale University Child Study Center, School of Medicine, New Haven, CT 06519, USA; alanskaufman@gmail.com

**Keywords:** Wechsler scales, WAIS-IV, federal judges, Supreme Court, IQ, intelligence, fluid reasoning, processing speed, crystallized knowledge, working memory, aging-IQ research, computerized adaptive testing, test construction

## Abstract

U. S. Supreme Court justices and other federal judges are, effectively, appointed for life, with no built-in check on their cognitive functioning as they approach old age. There is about a century of research on aging and intelligence that shows the vulnerability of processing speed, fluid reasoning, visual-spatial processing, and working memory to normal aging for men and women at all levels of education; even the maintained ability of crystallized knowledge declines in old age. The vulnerable abilities impact a person’s decision-making and problem solving; crystallized knowledge, by contrast, measures a person’s general knowledge. The aging-IQ data provide a rationale for assessing the key cognitive abilities of anyone who is appointed to the federal judiciary. Theories of multiple cognitive abilities and processes, most notably the Cattell-Horn-Carroll (CHC) model, provide a well-researched blueprint for interpreting the plethora of findings from studies of IQ and aging. Sophisticated technical advances in test construction, especially in item-response theory and computerized-adaptive testing, allow for the development of reliable and valid theory-based tests of cognitive functioning. Such assessments promise to be a potentially useful tool for evaluating federal judges to assess the impact of aging on their ability to perform at a level their positions deserve, perhaps to measure their competency to serve the public intelligently. It is proposed that public funding be made available to appoint a panel of experts to develop and validate an array of computerized cognitive tests to identify those justices who are at risk of cognitive impairment.

U. S. Supreme Court justices and other federal judges are, effectively, appointed for life, without checking potential cognitive decline. Yet, there is a century of research on aging and intelligence e.g., (Hertzog 2020; Willoughby 1927), with empirical findings showing the vulnerability of abilities like reasoning and speed to the normal aging process (Salthouse 2014a). Even the maintained ability of crystallized knowledge has been shown to decline steadily after about age 70 or 75 in cross-sectional studies controlled for educational attainment e.g., (Kaufman and Horn 1996); in cohort-sequential studies that blend cross-sectional and longitudinal methodologies (Schaie 2012); and in quasi-longitudinal studies, which follow different samples of adults over time based on their year of birth (Lichtenberger and Kaufman 2013). These findings occur for men and women, for all educational levels, and across generations (Kaufman et al. 2016). 

## 1. The Problem

I believe that the overwhelming consistency in the literature on aging and intellectual decline should call into serious question American practices concerning the length of judicial appointments. Issues associated with the maintenance of cognitive abilities across the lifespan have been prominent for generations and are noteworthy in recent times when powerful members of the Senate and House of Representatives, presidential candidates, and presidents in both parties have been elderly, and controversy persists regarding the wisdom of lifetime appointments for federal judges. 

In fact, Article III of the Constitution, which spells out the terms for all Federal Court justices (Supreme, Circuit, and District), specifies that they “shall hold their Offices during good Behaviour”. According to Brian Hays, JD (Personal communication, 8 October 2021): “Though not explicitly stated in the Constitution, that clause has come to be interpreted to mean lifetime tenure for judges”. The Founding Fathers might have been thinking about corruption or criminal behavior; however, a diminished capacity to solve problems and make decisions would seem to fit into the Article III guidelines. 

## 2. The Theory 

The accumulated literature on aging and intellectual development, though often not specifically inspired by theory (Hertzog 2020; Salthouse et al. 2008; Schaie 1983a, 1983b, 2012), is easily interpretable from three interdependent theoretical foundations: Raymond Cattell and John Horn’s developmental theory of fluid and crystallized intelligence (*Gf*-*Gc* theory; Carroll 1963; Horn and Cattell 1967); Horn’s (1989) subsequent expansion and elaboration of the two-pronged *Gf-Gc* theory to include a multiplicity of abilities; and John Carroll’s (1993) extensive synthesis of factor-analytic research on cognitive abilities into a three-tiered hierarchical model. 

Horn’s and Carroll’s different approaches yielded essentially the same eight to 10 Broad Abilities (the middle tier in Carroll’s model), expanding the number of abilities from the original two—Fluid Reasoning (*Gf*) and Crystallized Knowledge (*Gc*)—to an array that includes Processing Speed (*Gs*), Short-term Memory (*Gsm),* Visual-Spatial Processing (*Gv*), and Long-term Storage and Retrieval (*Glr*), among others. Today, top CHC experts believe that research supports perhaps as many as 20 broad abilities, including Working Memory Capacity (*Gwm*), previously categorized as a narrow ability in the bottom tier of Carroll’s hierarchy (Schneider and McGrew 2018). 

The latest version of the CHC model is illustrated nicely in a figure presented by Schneider and McGrew (2018, p. 75). The figure displays 17 Broad Abilities, three of which are unconfirmed e.g., *Gh*, or Tactile Processing. It depicts three intersecting circles under the heading Acquired Knowledge: *Gc*, *Gq* (Numeracy); and *Grw* (Literacy). *Gf* and *Gwm* are depicted as slightly intersecting circles under the heading *Controlled Attention*, along with a third circle, *Gs*. Quite clearly, the latest iteration of CHC theory emphasizes the complexity of crystallized knowledge, the interaction of the diverse set of Broad Abilities, and the need to integrate these abilities to solve problems. 

Although Carroll believed in an overarching general ability or *g* factor (the top tier in his model), Horn defiantly did not. Nonetheless, the three bodies of research were synthesized in the mid-1990s into a single theory, the Cattell-Horn-Carroll (CHC) model of intelligence (Schneider and McGrew 2018). 

## 3. The Research

As noted, CHC theory provides a theoretical foundation for interpreting literature on intelligence and aging. Salthouse (2010) summarized the results of a wealth of *cross-sectional* aging data as follows:

On one hand, there is increase, at least until people are in their 60s, for measures representing products of processing carried out in the past, such as vocabulary or general information in which the relevant acquisition occurred earlier in one’s life. On the other hand, there is nearly linear decline from early adulthood on measures representing efficiency or effectiveness of processing carried out at the time of assessment, usually involving manipulations or transformations of abstract or familiar material (p. 754). 

These consistent findings from cross-sectional investigations of the maintenance and vulnerability of specific cognitive abilities were observed more than a half-century ago (Birren and Morrison 1961) and called the classic aging pattern by Botwinick (1977). These results generalize to a variety of instruments via an array of methodologies, such as quasi-longitudinal studies (Kaufman 2001; Lichtenberger and Kaufman 2013; Salthouse 2014a) and a merger of cross-sectional and longitudinal techniques (*cohort-sequential*; Schaie (1983b, 2012)). Baltes et al. (1999) define the two basic curves of intellectual development as the consistent negative changes in cognitive mechanics versus the preservation of *pragmatics*.

## 4. The “Classic Aging Pattern” Based on Cross-Sectional Analyses

**Figure 1:**
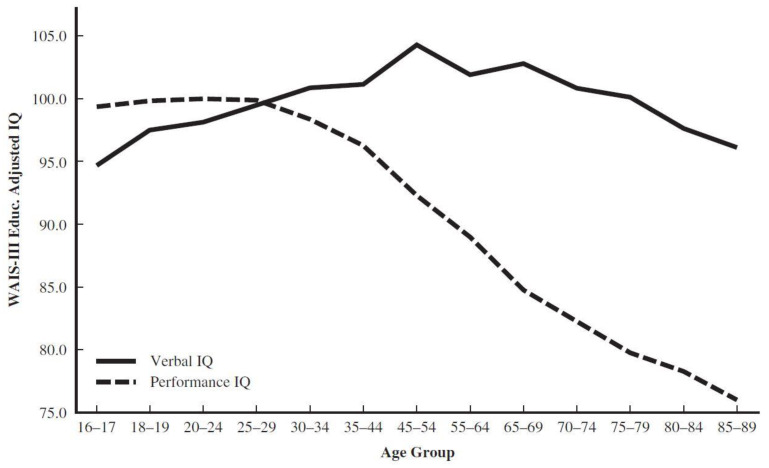
*Cross-Sectional Data—Age Patterns of Maintained* Vs. *Vulnerable Abilities:* Mean “Reference Group” (Ages 20–34) WAIS-III Verbal and Performance IQs, by Age, for Adults Ages 16–17 to 85–89 Years, Adjusted for Educational Attainment (Values for Ages 16–19 are Unadjusted). Source: Data are from Kaufman (2001). Figure 1 appeared in Lichtenberger and Kaufman’s (2013) *Essentials of WAIS-IV Assessment (2nd ed.), published by John Wiley, on page 264 as Figure 7.3, on page 264*. Reproduced with the permission of the publisher. Copyright © 2013 by John Wiley & Sons, Inc. All rights reserved. Published by John Wiley & Sons, Inc., Hoboken, New Jersey. Published simultaneously in Canada.

Figure 1 displays the classic aging pattern with cross-sectional data on the Verbal IQ (V-IQ) and Performance IQ (P-IQ), for ages 16 to 89 years, on the Wechsler Adult Intelligence Scale—Third Edition (WAIS-III; Wechsler (1997). To enable comparisons across age groups, IQs for all adolescents and adults are compared to a reference group of 20–34-year-olds, instead of to their age peers. To try to limit the cohort effects that are inherent in cross-sectional analyses, mean standard scores of the adult portion of the sample are adjusted for educational attainment (data from Kaufman (2001)). Based on Cattell and Horn’s *dichotomous Gf-Gc* theory, V-IQ corresponds to *Gc* and P-IQ to *Gf*, where the former reflects a person’s acquisitions and knowledge since childhood and the latter measures the speed and accuracy of solving new problems. 

When relying on modern CHC theory and its plethora of cognitive abilities—rather than on the two-pronged *Gf-Gc* model that preceded it—V-IQ and P-IQ have been shown by CHC researchers to be crude amalgams of multiple abilities. V-IQ measures *Gc*, number ability, and memory (including working memory), whereas P-IQ assesses reasoning, speed, visual-spatial processing, and dealing with novelty. The multi-factored nature of V-IQ and P-IQ is also evident from the statistically sophisticated array of investigations with different methodologies, tests, and samples conducted by Salthouse and his colleagues for more than a generation e.g., (Craik and Salthouse 2008; Salthouse 1985, 1998, 2010; Salthouse et al. 2008). 

Even so, the early Cattell-Horn distinction displayed in Figure 1 is revealing. The ability to rapidly solve new problems peaks in the late 20s, plateaus briefly, and declines at the rate of about five IQ points per decade, starting in the mid-40s. By contrast, facts and skills learned via schooling and acculturation increase to about age 60 (as long as education is controlled) before beginning its inevitable decline. 

The most recent Wechsler adult scale, the 4th edition (WAIS-IV; Wechsler (2008)), relies solely on four Indexes (plus a Full Scale IQ) that align nicely with CHC theory and with the four abilities that Salthouse and colleagues have investigated, primarily using the hand-picked 16-subtest individually-administered battery, labeled the VCAP (Virginia Cognitive Aging Project; Salthouse et al. (2008)). The four VCAP abilities are essentially the same as those measured by the four WAIS-IV Indexes (Salthouse 2009).

In fact, neither Wechsler’s adult scales nor the VCAP were built from a theory-based foundation, but CHC theory provides a shared vocabulary that communicates effectively to researchers and clinicians alike (McGrew 1997) and provides a useful framework for interpreting the differential age changes on the four well-researched abilities.

### 4.1. Longitudinal Analyses of Age Changes across the Lifespan

It is ideal to base research findings regarding aging and intelligence on longitudinal (rather than cross-sectional) studies, where the same individuals are tested over their lifetime (Hertzog 2020; Schaie 1983a, 2012). With many longitudinal studies, however, the declines in reasoning and speed are obscured because of methodological issues, especially selective attrition (low-scoring adults tend to drop out) and the profound practice effect and progressive error, particularly on nonverbal and speeded tasks (Lichtenberger and Kaufman 2013; Salthouse 2014b). Those issues compromise interpretation of data from most longitudinal studies, such as the well-known Duke longitudinal studies (Siegler 1983), in which adults were tested as many as 11 times on the original Wechsler Adult Intelligence Scale (WAIS; Wechsler (1955)).

Nonetheless, excellent longitudinal investigations of cognitive abilities across the lifespan have been conducted (Hertzog 2020), most notably Schaie’s (2012) extensive series of cohort-sequential investigations in Seattle, along with other innovative studies in Victoria, Canada (Hultsch et al. 1998); Long Beach, CA (Zelinski et al. 2009); and northern Sweden (Ronnlund et al. 2005). However, definitive interpretation of patterns of decline in different cognitive abilities is contaminated in these studies (Lichtenberger and Kaufman 2013; Salthouse 2009). Statistical modeling has been used to attempt to correct for this contamination, but the effectiveness of these models is controversial (Salthouse 2009; Schaie 2009). 

**Schaie’s Investigations.** Another disadvantage of Schaie’s (1983b, 2012) studies is the instrument used in all phases, namely the old group-administered Primary Abilities Test (PMA; Thurstone and Thurstone (1949)) designed for children and adolescents. Ultimately, Zelinski et al. (2009) restandardized the PMA for adults without revising it, starting in 1978. That adult standardization served as the initial impetus for the Long Beach Longitudinal Study, resulting in the development of the STAMAT (Schaie-Thurstone Adult Mental Abilities Test; Schaie (2012)).

Because the STAMAT was not revised, it had the advantage of allowing direct cross-validation of Schaie and colleagues’ results with its five subtests—Verbal Meaning, Spatial Orientation, Inductive Reasoning, Number, and Word Fluency. The disadvantages, however, were considerable. As noted, the original test was developed for children and adolescents, not adults, and lacked difficult items. All tasks, even Verbal Meaning (a measure of *Gc*), were timed to balance the low ceilings; that enabled processing speed to affect the patterns of decline on all five tasks (Hertzog 2020). In fact, all tasks showed similar patterns of decline into old age, although Hertzog (1989, 2020) believed the decline for the *Gc* subtest was an artifact of its speeded nature.

**Brief Overview of Findings from Longitudinal Studies.** Overall, the best-designed longitudinal studies identified, “curvilinear patterns of average age changes from the period of midlife through old age, with an acceleration in the rate of aging effects on fluid intelligence, episodic memory, and spatial visualization… after age sixty-five” (Hertzog 2020, p. 186). The studies also demonstrated substantial cohort effects (i.e., differences in age changes based on year of birth) on tests of *Gf* and *Gv*, but not on *Gs*, and perhaps not on *Gc*—even though *Gc* would seem to be the best candidate for significant cohort effects (Hertzog 2020).

### 4.2. Quasi-Longitudinal Investigations of Lifespan Age Changes

**Figure 2:**
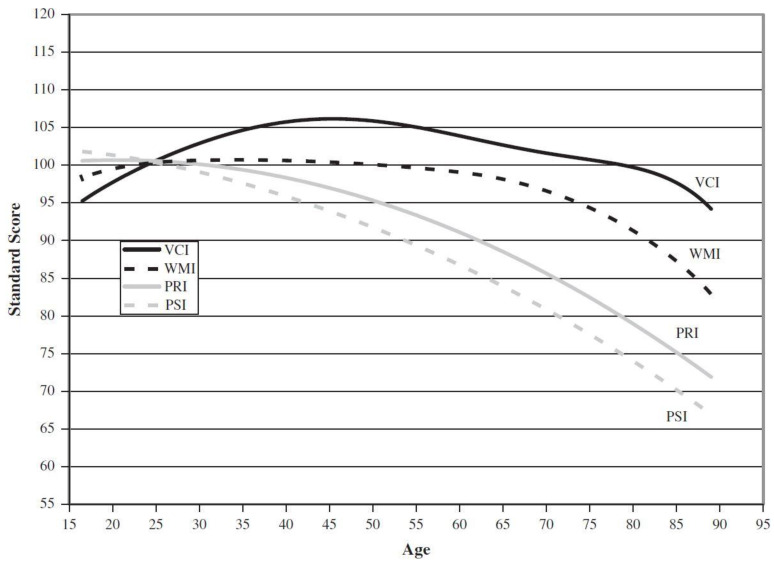
*Quasi-Longitudinal Data—Age Gradients for the Four WAIS-IV Indexes* Based on Mean Scores Earned by the 11 Cohorts when Tested in 1995 on the WAIS-III and in 2007 on the WAIS-IV. Source: Data are from Lichtenberger and Kaufman (2009). Analysis results from the Wechsler Adult Intelligence Scale—Fourth Edition (WAIS-IV). Copyright © 2008 by NCS Pearson, Inc. Reproduced with permission. All rights reserved. “Wechsler Adult Intelligence Scale” and “WAIS” are trademarks, in the United States and/or other countries, of Pearson Education, Inc., or of its affiliate(s). Figure 2 appeared in Lichtenberger and Kaufman’s (2013) *Essentials of WAIS-IV Assessment (2nd ed.),* published by John Wiley, on page 288 *as* *Figure 7.7.* Reproduced with the permission of the publisher. Copyright © 2013 by John Wiley & Sons, Inc. All rights reserved. Published by John Wiley & Sons, Inc., Hoboken, New Jersey. Published simultaneously in Canada.

The methodological problems with longitudinal studies suggest the need to control the selective attrition, progressive error, and differential cohort effects. Consequently, quasi-longitudinal studies, which follow the same *age cohorts*, but not the same *individuals*, across generations, have advantages when examining patterns of gains and declines over time (Salthouse 2014b). Figure 2 displays these distinct aging patterns for the four Wechsler Indexes for ages 16 to 90 years, based on a quasi-longitudinal investigation of the WAIS-III and WAIS-IV (Wechsler 1997, 2008) that followed representative samples of the same age cohorts across generations. In order not to obscure age differences in these cohorts, the standard scores for *all* adults were based on a common norms group of young adults, rather than on separate age norms (Lichtenberger and Kaufman 2013).

In that study, the cognitive abilities of 11 age cohorts from the WAIS-III and WAIS-IV standardization samples were followed over a 12-year interval; for example, those born between 1966–1970 were about 27 when the WAIS-III was normed in 1995 and were about 39 when the WAIS-IV was normed in 2007; similarly, the 1926–1930 cohort averaged 67 and 79 years of age, respectively, at the two points in time. This procedure controls for cohort effects but requires statistical adjustment for instrument effects (3rd vs. 4th edition) and time-lag effects (cultural change).

As shown in Figure 2, the Verbal Comprehension Index, which measures *Gc*, a maintained ability, increases through late middle age before starting its decline. By contrast, the other three Indexes are vulnerable abilities, with processing speed (*Gs*, measured by the Processing Speed Index) peaking at age 16½. The Perceptual Reasoning Index, which measures both fluid reasoning (*Gf*) and visual-spatial processing (*Gv*), peaked in the mid-20s. All three of these abilities decline dramatically and steadily through old age. Adults ages 75 to 90, when evaluated against a young adult reference group, earn mean standard scores between the mid-60s and low 80s in reasoning and speed (more than 1 to 2 standard deviations below the mean). Most mean values for this age group land squarely in the range sometimes referred to as *Borderline Intellectual Functioning* (the 70–79 range), coined by Terman (1916) to denote the slippery slope between intellectual disabilities, “and the higher group usually classified as normal but dull” (p. 87). 

For the four oldest age groups in the quasi-longitudinal study depicted in Figure 2 (i.e., ages 59.5, 67, 72, and 77 years in 1995), the mean effect sizes of the 12-year change (1995 to 2007) are as follows: Processing Speed (0.56 *SD*); Fluid Reasoning/Visual-spatial ability (0.52 *SD*); Working Memory (0.40 *SD*); and Crystallized Knowledge (0.39 *SD*) (Lichtenberger and Kaufman 2013, Table 7.9). Based on Cohen’s (1988) suggested guidelines for interpreting effect sizes of difference scores, a value of 0.2 is considered small, with values of 0.5 and 0.8 denoting medium and large effect sizes, respectively. For 12-year intervals, declines are of small to medium effect sizes.

It is also of interest to examine cognitive declines over longer intervals. Based on Wechsler adult scale quasi-longitudinal data at three points in time (1953, 1978, 1995), changes over the 25-year span between 1953 and 1978 on P-IQ were examined for the four age cohorts, as were changes over the 42-year span (1953–1995). The age cohort born 1924–1933 was 24.5 in 1953, 49.5 in 1978, and 66.5 in 1995. The second cohort (1914–23) was 34.5, 59.5, and 76.5, respectively; the third (1909–13) was 42, 67, and 84; and the fourth (1904–08) was 47, 72, and 89. 

The decrease in adjusted mean P-IQ was very similar for each of the four cohorts over the 25-year interval, ranging from 0.77 *SD* to 0.9 *SD*, all large effect sizes. Over the 42-year interval, the values were a virtual constant, ranging from 1.47 *SD* to 1.53 *SD*, very large effect sizes (Lichtenberger and Kaufman 2013, Table 7.7). 

Though the Working Memory Index is also vulnerable to the effects of normal aging, the decline in working memory capacity (*Gwm*) is not as precipitous as the age-related decreases in reasoning and speed. These results demonstrate the separate age gradients for the CHC abilities studied extensively by Salthouse and colleagues and measured by the Wechsler Indexes. Further, the distinct age patterns shown in Figure 2 align closely with (a) results of other quasi-longitudinal studies of Wechsler’s scales over intervals of 25 years (1953–1978; Kaufman (1990)) and 17 years (1978–1995; Kaufman (2001)); (b) with findings from cross-sectional studies of an array of instruments over several generations (Kaufman et al. 2016; Salthouse 2009, 2010); and (c) with the results of Schaie’s (1983b, 2012) cohort-sequential investigations of the PMA and STAMAT. These consistent results indicate that a theory that allows for multiple abilities, such as CHC or Thurstone’s (1938) PMA model, is needed to explain the nuances of the cognitive aging process; a dichotomous model, such as *fluid-crystallized*, *Verbal-Performance*, *vulnerable-maintained*, or *cognitive mechanics-pragmatics* is too simplistic. 

### 4.3. Age Changes in Abilities for Different Levels of Education

These investigations of changes in intellectual abilities from late adolescence to old age evaluated *group* differences, to be sure, and the data are largely based on heterogeneous samples that span the wide range of intelligence from intellectual disabilities to extreme giftedness. By contrast, federal justices are a more elite, educated group, and the results of research on aging across the lifespan by occupation or education is voluminous and subject to varied interpretations. Studies of elderly eminent academics versus elderly blue-collar workers (Christensen et al. 1997)—and of old versus young Berkeley professors (Shimamura et al. 1995)—showed similar patterns of decline in old age. However, disputes reign. Some teams of investigators have concluded that higher education is “protective” against cognitive decline in old age e.g., (Arbuckle et al. 1998); others disagree (Anstey and Christensen 2000). 

More recent analysis of data from Wechsler’s adult scales supports two basic findings: (a) that more educated adults *outperform* less educated adults on all sorts of cognitive tasks, but (b) the *rate of decline* across the lifespan is essentially the same for all education levels. That finding was endorsed unequivocally on Wechsler’s scales, often considered the gold standard of intelligence, based on multiple regression analyses conducted on three generations of Wechsler’s adult scales (Kaufman et al. 2016).

### 4.4. Implications of the Body of Aging Research

Taken together, the declines in key cognitive abilities for the elderly shown in Figure 1 and Figure 2 impact a person’s executive functioning in areas such as judgment, decision-making, problem solving, concept formation, attention, memory, concentration, and planning ability (Flanagan et al. 2013, Table 3.6). This set of skills bears an intuitive relationship to the diverse kinds of real-life problem solving and effective processing of in-depth information required to be an effective, intelligent judge. Further, some researchers argue that coping with the challenges of complex occupations throughout adulthood, such as performing the job functions as a federal judge, helps maintain an adult’s cognitive abilities through old age (see Krampe and Charness (2018), for a review). If so, then judges who show substantial decrease in their intellectual abilities, despite the enhancing effect of their life’s work, are noteworthy. Decline in these abilities is likely to negatively affect their continued job performance. Further, there are a number of other tests that need to be considered for the proposed battery based on modern CHC theory (Schneider and McGrew 2018). Additionally, Ackerman’s (2018, 2020) compelling decades-long research on expertise provides an empirical foundation for developing a criterion measure for validating the new set of computerized tests.

One thing is for certain: The accumulated group data—as compelling as they are for adults in general, including highly educated adults—do not generalize to any particular individual at any specific point in their lifetime. For that information, each adult must be assessed on reliable and valid tests to determine their current levels of functioning in key cognitive domains.

## 5. The Proposed Solution

I propose that public funds be made available to set up a bipartisan team of Test Research and Development Experts (TRADE), composed of prominent test developers, psychological researchers and theorists, methodologists, statisticians, clinical neuropsychologists, neuroscientists, neurologists, psychiatrists, clinical psychologists, lawyers, retired judges, historians, political scientists, and software engineers: (a) to develop a computerized test battery to evaluate the cognitive abilities and academic skills of all federal judges (Supreme, Circuit, and District) who are either currently on the bench or newly appointed to it; (b) to develop a computerized test of judicial competence to serve as a criterion measure to validate the battery of cognitive tests; and (c) to follow the test development phase with a rigorous validation stage to determine the effectiveness of the new test battery for identifying those judges who may no longer be able to fulfill their duties intelligently. 

In the opinion of Thomas Dillon, PhD, who served in the Commerce and Energy Departments in the 1970s and 1980s during Republican and Democratic administrations: “The prospect of testing that could reliably assess the impact of aging on an individual’s intelligence opens up a spectrum of very exciting options for application to public service… I really do believe that the country is ripe for such an approach as the public views a federal government that appears to be rife with octogenarians” (Personal communications, 5 December 2020 & 15 October 2021).

### 5.1. SMART—A Government—Funded Precedent

There are precedents for research programs sponsored by the federal government. The U. S. Office of the National Director of Intelligence includes, among its programs, Strengthening Abstract Reasoning and Problem-solving (SMART; Kyllonen et al. (2019)). The goals of SMART are to develop tests for high-ability people and then try to validate interventions to improve the agents’ abstract reasoning and critical problem-solving ability. These goals are entirely consistent with the aims of the TRADE panel I am proposing. The defense department has been applying intelligence theory, including the CHC model, to enhance the cognitive abilities of their intelligence agents (Schneider and McGrew 2018). Why not also provide funding to identify those federal judges whose abilities might be declining?

### 5.2. Initial Roles of the TRADE Panel—Developing and Validating the Tests

Limited competence in crystallized knowledge, processing speed, fluid reasoning, and working memory casts doubt on a judge’s capacity to serve the public intelligently, or to continue in that service. However, that hypothesis must be validated before the tests are put to use, just as any new test requires validation before it is used for any type of decision-making. The panel is deliberately composed of an array of experts from diverse fields to allow preliminary discussions to be wide ranging and to blend expertise in psychological theory and research with expertise in legal and judicial matters. The dialogue among experts needs to address, and attempt to resolve, one fundamental question prior to any actual test development: Are the abilities proposed for the test relevant for the duties, responsibilities, and complex decision-making required of judges? 

Further, the test development team should investigate other abilities for inclusion in the new battery. The four abilities featured in Figure 2 have a broad base of research to support their characteristic patterns of growth and decline across the lifespan. But other well-researched abilities and academic skills also deserve consideration (Schaie 2012; Schneider and McGrew 2018), and should be discussed regarding the degree to which they are relevant to a judge’s competent job performance. These additional abilities would extend to real-world skills—such as wisdom, practical intelligence, overcoming first-level perceptions, inhibition, and adaptation to the environment (Gluck 2020; Kahneman 2011; Sternberg 2019, 2020)—that are not included in traditional IQ tests but are clearly pertinent to a judge’s capabilities, perhaps even more so than IQ or CHC abilities. 

The important question for the methodologists, psychological theorists, test developers, and software engineers on the expert panel to debate is the feasibility of developing reliable and valid psychometric tests of these clearly important aspects of intelligence. If the answer is even “maybe”, then the time and money should be budgeted during the test development phase to try to reliably measure one or more of these highly relevant domains. The ultimate goal of the program is the validity phase to determine if this new test can accurately tap into a judge’s capacity to remain on the bench for their lifetime. 

As noted, the extensive research on expertise conducted by Ackerman (2018, 2020) and his team offers insight into the development of a test of judicial knowledge to measure the facts and skills that legal experts deem essential to a federal judge’s competent performance on the bench.

### 5.3. Subsequent Roles of the TRADE Panel—Setting Up Guidelines for the Tests

The TRADE panel would identify the most robust statistical procedures to set the criteria for passing or failing the new battery of tests, based on analysis of a plethora of reliability and validity data gathered during the two phases of the research program; They would also decide what range of scores is deemed indeterminate, based on errors of measurement and patterns of strengths and weaknesses on the test battery. However, the data would be balanced by the expert opinions of panel members, especially those who specialize in psychometrics, clinical neuropsychology, the law, and the judiciary. There is no simple or absolutely correct way to set the cut scores. However, the cut score for each test needs to identify those with extremely poor functioning in that cognitive ability. This is high stakes testing and the outcome of the testing has vital consequences for the individual judge and for the federal judiciary.

Absent a consensus among the TRADE panel members, my recommendation would be to use a cut-score, for each separate test, that is 2 standard deviations below the mean, corresponding to a standard score of 70 and a percentile rank of 2. I would also suggest that a judge must score below that cut-score on at least two of the tests, perhaps three.

Further, all cut-off scores for the component tests would be banded by 95% confidence intervals to ensure that no one is penalized for missing the cut by one or two points. That same level of confidence is used to determine whether death row criminals are intellectually disabled, a diagnosis that would exempt them from capital punishment (i.e., an IQ range of 65–75 is applied, rather than a simple cut-off of 70) (Polloway 2015).

In capital punishment cases, no one is put to death based on a single test score. The criminal’s cultural opportunities, social-adaptive functioning, medical history, and family background all enter into the decision-making process. That same type of protection would be built in to the assessment of federal judges’ competency. Every judge would be evaluated with the same yardstick; that is to say, cut-off ranges for each test would not vary from judge to judge, regardless of individual circumstances. 

Nevertheless, there would be flexibility, not rigidity, in the process, and there would be built-in safeguards to ensure that no final decision is made without the opportunity for appeal. Those judges who are deemed, unequivocally, to fail the test battery would be asked to resign without fanfare; but they would be allowed an appeal. That appeal would be automatic whenever equity is at issue—for example, for judges from non-mainstream backgrounds, or for those who claim they need some kind of accommodation due to physical, psychological, cultural, or linguistic factors. 

Those whose scores are too close to call, or who appeal the test results, would be given a thorough neuropsychological evaluation by a top-rated clinical neuropsychologist. The examiner would be selected randomly from a pool of expert clinicians, pre-approved by the bipartisan TRADE panel. The chosen examiner would conduct thorough one-on-one evaluations—using state-of-the art clinical measures of intelligence, achievement, personality, and memory—to identify those judges with dementia or any other type of cognitive impairment that would compromise their capacity to render intelligent decisions. Those judges who opt to appeal their requested resignation would be assessed with accommodations made to ensure equitable assessment for all judges from all backgrounds.

Clinical neuropsychologists who believe that the judge’s low cognitive test scores are primarily the result of mental illness, a physical illness, traumatic brain injury, Alzheimer’s, a personality disorder, and the like, are obligated to refer the judge for further evaluation. That referral might be to a clinical psychologist, neurologist, psychiatrist, gerontologist, or any other relevant professional who might provide a more definitive diagnosis. All of these professionals would either be TRADE panel members, or approved by that bipartisan panel.

The TRADE panel would determine whether to assign weights to the test results versus the psychological or medical diagnosis in deciding the judge’s fate—or whether to consider the professional’s verdict final and, perhaps, not subject to appeal. The panel would be responsible for determining the degree to which the public would be informed about any or all of the proceedings, either before, during, or after a decision has been made about a judge’s tenure; it would also set the protocol for how frequently a federal judge would be assessed to determine their competency; whether elderly judges should be tested more often than young or middle-aged judges; how much money to compensate each judge for the time it takes for the computerized tests, and (when applicable) for the thorough neuropsychological evaluation; and how to deal with uncooperative judges who refuse to take the test or do not give full effort during the computerized administration. In general, the panel would set all guidelines concerning test content, test security, privacy, protection from hackers, dissemination of data, and the like.

If the computerized tests prove to be valid in detecting cognitive impairment in sitting and newly appointed federal judges, and gain public approval, then the government-sponsored TRADE panel would have worldwide implications. A successful program in the U. S. would provide a test research and development prototype for other world democracies.

### 5.4. Questions That the TRADE Panel Must Address to Secure and Maintain Funding 

In order for the TRADE program to obtain initial funding, and to continue being awarded that governmental support, the proposal for its implementation must answer, with scientific rigor, some key questions about the rationale for its proposed content and features; and whether existing technology is capable of developing reliable and valid computerized tests. Some of the more important of these questions are addressed in the sections that follow.

### 5.5. Isn’t a Test of Crystallized Knowledge Sufficient?

Some might contend that only one test is needed for evaluating a judge’s intellectual capacity, namely *Gc*. They might argue that high scores on tests of crystallized knowledge should be adequate to justify the retention of a judge, regardless of their levels of functioning on other abilities such as *Gf* and *Gs*. However, there are compelling arguments against that contention.

**Gc Does Not Even Provide an Adequate Measure of Acquired Knowledge.** First, *Gc*, as measured on IQ tests, assesses a person’s general knowledge base and language skills (Ackerman 2020). Schneider and McGrew’s (2018) overview of the modern CHC model indicates four key components of Acquired Knowledge: (a) *Gc,* defined as Verbal Comprehension + General Knowledge; (b) *Gq*, or Numeracy; (c) *Grw*, or Literacy; and (d) tests of domain-specific knowledge (*Gkn*), such as expertise in jurisprudence, biology, or opera. *Gc*, by itself, is not a thorough measure of a person’s knowledge accrued via formal and informal education, training, job experience, and acculturation. 

Based on an accumulation of research studies over the past 35 years, Ackerman’s (2018, 2020) team has provided much evidence that *Gc*, as measured by IQ tests, greatly underestimates the knowledge base of adults; it excludes the specific areas of expertise that each adult has personally acquired through their unique experience and practice. Different types of *Gc* may demonstrate distinct cross-sectional aging curves, depending on the type of knowledge being measured (Ackerman 2020; Hertzog 2020). Ackerman (2000, 2020) and his colleagues e.g., (Beier and Ackerman 2005, in particular, have extensively researched the construct of expertise; they have studied the specific kinds of crystallized knowledge that adults learn during their lifetimes, often related to their occupations, interests, and hobbies. This type of expert or *declarative* knowledge differs from the kinds of conventional *Gc* measured by tests of information and vocabulary, such as Wechsler’s or the Woodcock-Johnson’s adult scales. 

**Additional Measures of *Gc*.** There is, therefore, a strong rationale from both theory and applied research to support the inclusion of at least two separate measures of acquired knowledge in the test battery: traditional *Gc* as measured by IQ tests; and a *Gkn* test that assesses the declarative and procedural knowledge that is demonstrated to be a valid measure of a judge’s specific set of facts and skills that are deemed essential to being competent and successful in performing the varied roles of a federal judge. 

**Numeracy and Literacy.** Quantitative Knowledge (*Gq*) is another CHC ability that merits inclusion in the new test battery. *Gq* is “*the breadth and depth of declarative and procedural knowledge related to mathematics*… For a complex society to function, most adults must have mastered core numeracy skills… To provide public services and to guide public policy, a sizeable proportion of the population must also understand the basics of algebra, geometry, and statistics” (Schneider and McGrew 2018, p. 123, italics in original). A test of *Gq*, usually found to be a vulnerable ability, has been studied extensively by Schaie (2012) and other researchers e.g., (Kaufman et al. 1996; Kaufman et al. 2008; Kaufman et al. 2009; McArdle et al. 2002); *Gq* should be considered for the new test battery.

So, too, should a test of reading comprehension, to round out the measures of acquired knowledge. Reading decoding and reading comprehension (both aspects of *Grw*) have been shown to be maintained and vulnerable, respectively (Kaufman et al. 2009). Reading comprehension is especially important to consider for inclusion because of the heavy reading burden of judges (even with a roomful of clerks to carry much of the reading load); the fact that it requires a good amount of inferential reasoning; and its vulnerability to the aging process. To increase the relevance of the reading comprehension test, the passages would reflect the kinds of documents a judge would ordinarily be expected to read, such as law briefs, previous Supreme Court decisions, case files, *ex parte* decisions, and the like.

### 5.6. Won’t Multiple Tests of Acquired Knowledge Suffice to Evaluate a Judge’s Competency?

The short answer is “No”. Even reliable and valid measurement of *Gc*, *Grw*, *Gq*, and *Gkn* are not sufficient to evaluate a federal judge’s competence to stay on the job for life. Assessing multiple aspects of acquired knowledge, no matter how thorough the tests, is simply not enough to make an informed decision. In order to apply a judge’s range of acquired knowledge to solve complex real-world problems and to make high-level decisions, they need intact fluid reasoning. 

**Reciprocal Relationship Between *Gc* and *Gf*.** Importantly, and historically, *Gc* and *Gf* have been shown to be interdependent e.g., (Beier and Ackerman 2005; Carroll 1987; Hambrick and Engle 2002). To summarize that reciprocity: 

Fluid reasoning creates new knowledge, and via memory, knowledge accumulates; today’s fluid insights become tomorrow’s crystallized knowledge… [;] crystallized knowledge provides conceptual structures on which new fluid insights are likely to occur. … This is one of the reasons why experts absorb new information related to their discipline more quickly than information unrelated to their expertise (Schneider and McGrew 2018, p. 92).

## 6. Interdependence of *Gf*, Working Memory Capacity, and Processing Speed

Further, *Gf* is heavily dependent on both *Gwm* and *Gs*. To perform well on tests of *Gf*, success depends on one’s ability to handle and integrate abstract relationships between increasingly complex sets of stimuli. To juggle the elements of a problem, and to keep the key elements in one’s immediate awareness, require excellent working memory capacity, namely “*the ability to maintain and manipulate information in active attention*” (Schneider and McGrew 2018, p. 97, italics in original). Quite evidently, *Gf* seems to depend quite heavily on *Gwm* (Shipstead et al. 2014).

*Gs* “*is the ability to control attention to automatically, quickly, and fluently perform repetitive cognitive tasks* (Schneider and McGrew 2018, p. 108, italics in original). Speed is not particularly important in the early, learning phases of skill acquisition—at least compared to *Gf* and *Gc*—but it is an essential predictor of skilled performance once people have learned the basic task and are seeking to develop expertise (Schneider and McGrew 2018). In view of the very rapid decline of *Gs* with increasing age, the assessment of the ability to control attention and process information automatically emerges as a potentially important test to evaluate a federal judge’s continued success on the job, particularly in a rapidly changing society.

### 6.1. Should Fluid Intelligence and Visual-Spatial Reasoning Be Separate Tests?

Salthouse and his colleagues originally studied five abilities, not four, when they began their research program; visual-spatial reasoning (*Gv*) and *Gf* were analyzed as separate abilities. However, Salthouse et al. (2008) ultimately merged *Gf* and *Gv* into a single ability, labeled *Fluid Reasoning*, because *Gf* and *Gv* correlated highly and displayed the same vulnerability to aging. Yet, for evaluating a judge’s continued ability to perform at a high level, *Gv* could easily be a stand-alone test that differs from “pure” *Gf* in the kinds of problems to be solved. Schaie (2012) and Zelinski et al. (2009), in fact, evaluated *Gf* (Inductive Reasoning) and *Gv* (Spatial Orientation) as separate measures in their comprehensive blend of cross-sectional and longitudinal investigations. 

Wechsler’s adult scales across generations are also noteworthy regarding the separation of *Gv* from *Gf*. These two CHC abilities have historically been tethered in Performance IQ ever since Wechsler (1939) published the Wechsler-Bellevue Intelligence Scale. That amalgam continued with the publication of the WAIS (Wechsler 1955) and its revisions (Wechsler 1981, 1997, 2008)—including its 4th edition—as either P-IQ (see Figure 1) or the Perceptual Reasoning Index (see Figure 2). The blending of *Gf* with *Gv* characterized the Wechsler Intelligence Scale for Children (WISC; Wechsler (1949)), as well, through the 4th edition (Wechsler 2003).

By contrast, the 5th editions of Wechsler’s scales yield five—not four—primary indexes with *Gv* and *Gf* measured as distinct abilities (Wechsler 2014, 2021). The separation of these two problem-solving abilities is also featured in other popular clinical tests of intelligence (Kaufman and Kaufman 2018; Schrank et al. 2014), consistent with modern CHC theory (Flanagan et al. 2013; Schneider and McGrew 2018). 

There is clearly justification from empirical research and clinical assessment for the TRADE panel to consider separate measures of *Gf* (abstract reasoning and logical thinking; e.g., Raven’s matrices) and *Gv* (visual-spatial problem-solving; e.g., Wechsler’s Block Design). A separate *Gv* test also makes practical sense. The ability to visualize stimuli in two- and three-dimensions, and to mentally manipulate them in space, is potentially a key skill to assess in an era that relies heavily on PowerPoint slides, graphic presentations, and a plethora of complex visual stimuli that predominate on an array of websites. 

However, the results of test development and validation research conducted by the proposed TRADE panel would need to determine whether separate tests of *Gf* and *Gv* are warranted. Similarly, the panel would need to determine precisely which tests, and how many, should be included in the test development phase of the research program and, ultimately, how many to include in the final test battery after the results of the validation phase have been thoroughly analyzed and cross-validated.

**The Proposed Test Battery.** Based on research, theory, and real-life problem solving, I believe the following eight tests should be proposed to the panel for consideration: Crystallized Knowledge (*Gc*), Fluid Reasoning (*Gf*), Working Memory Capacity (*Gwm*), Processing Speed (*Gs*), Visual-Spatial Reasoning (*Gv*), Expertise in Jurisprudence (*Gkn*), Reading Comprehension (*Grw*), and Quantitative Knowledge (*Gq*). Further, tests of adaptive constructs such as wisdom, adaptation to the environment, practical judgment, and inhibition, and should be pursued vigorously for possible inclusion. 

### 6.2. Should There Be a Global Score? 

I also would propose that no global IQ or composite score should be computed, a specious concept at best for tests that produce unique aging patterns across abilities and adult age groups, as displayed in Figure 1 and Figure 2. Based on data from the Woodcock-Johnson—Revised (Woodcock and Johnson 1989), McArdle et al. (2002) concluded that any model of changes in ability with age that relied only on *g* is too simplistic and misses the complexity of growth and decline over the lifespan.

### 6.3. Does Research Support the Use of Cognitive Tests to Predict Job Performance?

IQ has been correlated to job performance, usually supervisor ratings, for decades and decades. Literally hundreds of studies conducted through the 1960s yielded unimpressive validity coefficients in the 0.20 to 0.30 range, with considerable variability (Ghiselli 1973). Subsequent researchers reanalyzed the same data, this time correcting the values for a variety of so-called statistical artifacts such as attenuation and restriction of range, thereby doubling the validity coefficients to about 0.50 e.g., (Hunter and Hunter 1984; Schmidt and Hunter 2003). Values in the 0.50 to 0.60 range have since been consistently reported for new samples, including workers in European countries such as Great Britain (Bertua et al. 2005) and Germany (Lang et al. 2010).

This array of studies has been praised as offering widespread support for the validity of IQ as the best predictor of job performance. Some have been lavish in their praise: “Not relying on it [i.e., measured cognitive ability] for personnel selection would have serious implications for productivity. There is no getting away from or wishing away this fact” (Ones et al. 2005, p. 450). 

However, the uncritical high praise commonly given to the consistent results of a great many meta-analyses, does not stand up to careful scrutiny. Critical study-by-study interpretation of the methodologies are sobering and compelling e.g., (Richardson and Norgate 2015). Many criticisms are right on target: (a) correction for attenuation, that is to say for the fact that tests are not perfectly reliable, is neither standard practice nor justifiable; (b) supervisor ratings are notoriously unreliable; (c) meta-analyses have merged data on dozens of IQ tests, aptitude batteries, tests of working memory, and the like as if they are all comparable measures of *g*; and (d) many studies are from the military, which may not generalize to the typical workplace. 

The literature, therefore, offers only mild support for the use of cognitive tests to predict job performance. However, the serious criticisms of previous studies can be addressed. The use of general intelligence as a predictor defies contemporary theory and practice, both of which tend to focus on multiple cognitive abilities. Further, CHC theory and research provide rationales for measuring an array of specific abilities that are likely to be related to the complex job functions of a judge. And, perhaps most importantly, it should be feasible for the TRADE panel to develop a reliable and valid criterion of a judge’s many job functions.

### 6.4. What Criterion Would Be Used to Evaluate the Validity of the Tests?

One of the eight proposed tests would provide an excellent, reliable criterion to evaluate the validity of the other seven tests—namely, the measure of *Gkn* that assesses expertise in jurisprudence. The lawyers, retired judges, political scientists, and historians on the research panel would contribute to the development of this specialized test of judicial expertise. Content would include knowledge and skills specific to jurisprudence, acquired by both procedural memory and episodic memory; the test would be targeted for all judges—whether federal, state, or local—but its primary focus would be at the federal level. Whereas the relevant members of the TRADE panel would assist in formulating the test’s content, the main thrust for developing and validating this specialized test of *Gkn* would be true experts. In particular, I would recommend that current and former law clerks for federal judges would be the ideal experts for identifying the range and breadth of test content. They will have had firsthand knowledge of the extensive sets of facts and skills necessary to perform the complex and varied job functions of a federal judge. These esteemed professionals would be asked to participate in a structured interview to help provide the essential content for the criterion measure of judicial expertise. TRADE panel members would develop the survey questions and conduct the interviews. The validity of the *Gkn* test itself would derive from the unparalleled authority of the law clerks, and from statistical analyses e.g., (exploratory factor analysis, confirmatory factor analysis, logistic regression analysis) conducted during the development and validation of all tests in the new battery. 

Regarding federal law clerks, Brian Hays, JD (Personal communication, 9 October 2021), offered the following regarding the potential number of clerks in the pool:

My friend and classmate, Jim Bird, clerked for Judge Skelly Wright of the DC Circuit Court of Appeals in 1977–78, then went on to clerk for Supreme Court Justice William Brennan in 1978–79. He said that in a typical year there are 30 to 37 clerks for Supreme Court Justices. The number of living Supreme Court clerk ‘alumni’ is therefore perhaps 1000, but no more than 1500. Further, there are around 800 federal judges in the lower courts (Circuit and District) and most of the time, these judges also have clerks. Altogether, the pool of living law clerks probably exceeds 10,000 and might be closer to 20,000. A number of former federal clerks stay in touch. Jim went to a reunion right before the pandemic.

But law clerks are only one of many groups needed to develop and validate the measure of judicial expertise. Samples with differing levels of knowledge about judicial procedures would be tested, for example, lawyers, accountants, physicians, politicians, retired judges, political science professors, and active judges at the state or local level. Test scores on the measure of *Gkn* for the diverse samples would be compared; so too would validity coefficients for each sample to help establish convergent-discriminant validity.

### 6.5. Would Judges Who Earn High Scores on the Criterion Measure Be Required to Take the Remaining Cognitive Tests?

No, that would defy logic. The test of judiciary expertise would be the first test administered. Judges who “pass” the criterion should not be required to take any of the predictors of that criterion. If we have the answer, we don’t need to ask the question. 

The challenge is to determine the appropriate cut-off point to identify competency. Again, expert input, especially from current and former federal law clerks, would be instrumental in making that decision. The validation samples for the *Gkn* test would provide empirical input to blend with expert opinions. In particular, validation samples comprised of retired federal judges, current or retired state and local judges, legal experts on judiciary matters, and so forth, might be instrumental in helping set the cut score. Ideally, that cut score, banded by a 95% confidence interval, would determine which federal judges would be required to take the remaining cognitive tests.

### 6.6. What Kind of Norms Should Be Used to Determine a Person’s Scores?

By convention, every individual’s scores on IQ tests are based on their age peers. Nine-year-olds are compared to other 9-year-olds to compute their IQs; 17-year-olds are compared to others the same age; 22-year-olds are compared to their age peers (20–24); and so forth through the lifespan with the IQs of 88-year-olds compared to a group of 85–89-year-olds. 

For these new tests, I would propose to the TRADE panel that conventional standardization techniques should be abandoned. I favor basing everyone’s standard scores on stratified random samples of all adults (from young adulthood through old age) rather than on the performance of their age mates. The long-time clinical practice of basing an adult’s scores on their age peers obscures the effects of aging and is inconsistent with the fact that adults, in the real world, compete with each other, regardless of age. 

Wechsler made the decision to base children’s and adults’ IQs on their age-mates when he first published the Wechsler-Bellevue Intelligence Scale (Wechsler 1939) for ages 7 to 69 years. That decision has always made total sense for children, adolescents, and even young adults, all of whom are undergoing rapid intellectual development and are steadily gaining knowledge from elementary school, high school, and perhaps college. But that decision has never made sense for adults who are striving to enter graduate schools or technical schools; who are running for public office; who are seeking entry into a craft; who are applying for an industrial position or a training program or are competing for a promotion while climbing the business ladder; or who are actively engaged in most any group activity within a competitive society. 

Wechsler (1939) added to the confusion by basing scaled scores, on the 10 or so subtests, on a reference group of young adults (ages 20–34) for *all adults* regardless of age; but then he opened up a can of worms when he opted to base their IQs on their age peers. For many elderly individuals, the subtest scaled-score profile might reveal scores averaging 7 or 8, when compared to the reference group of young adults (16th to 25th percentile); but alongside those scaled scores would be IQs that ranked them at the 50th or 60th percentile, based on the performance of their age group.

This discrepancy between scaled scores and IQs was confusing to clinicians, especially when trying to explain the results to family members or educational and medical personnel at feedback conferences. Wechsler’s decisions about reference groups were made more than 80 years ago and were arbitrary; yet that practice has endured to the present day. 

Certainly the competency of federal judges is relative to other judges, whether they are 45 or 75; and the standard of competency does not shift for elderly judges. The research panel would make the ultimate decision of the best type of norms to use and what age range to include in an all-adult sample. My vote, if I had one, would be to base scores—for adults of every age—on a reference group that included a wide age range, such as all adults ages 21 to 99 years (or perhaps 25–99).

### 6.7. Do We Have the Technology to Develop the Proposed Computerized Tests?

We absolutely do possess the technology in the 21st century to develop and validate the ambitious computerized series of tests proposed in this paper. Coinciding with the accumulated literature on aging, intelligence, expertise, and CHC theory has been the sophisticated technical advances in test construction over the past two generations, most notably in (a) item-response theory (IRT), such as the Rasch-Wright latent-trait model (De Ayala 2009; van der Linden and Hambleton 2015); (b) computerized-adaptive testing (CAT; Wainer (2015)), that is to say, digitally administered tests that tailor each set of items to each person, based on their success or failure on the previous item; (c) methodologies for detecting item bias—differential item functioning (DIF; Holland and Wainer (2015); Zwick et al. (1995)); these statistical procedures help weed out items that are unfair to a particular subsample; (d) protocols for developing large banks of items for IRT-CAT designs to permit the continuous standardization of tests (van der Linden and Glas 2010); and (e) pertinent statistical and methodological Rasch-based IRT analyses, developed specifically from longitudinal data on vocabulary and memory, to deal with repeated constructs in developmental studies (McArdle et al. 2009). All of these advances make it feasible to develop CATs for the express purpose of assessing the cognitive abilities of all federal judges serving on the bench, or appointed to it, at any given point in time. 

**Item Banks, Continuous Norming, and IRT-CATs.** Literally tens of thousands of items could be developed for the item bank, enabling the tests to be continuously revised and re-standardized to partially neutralize practice effects and eliminate the Flynn Effect (Flynn 1987, 2020). Computerized tests could be administered to thousands of Americans per month if funded by the government. Data from stratified random samples of adults could be added to the normative base and the norms could be updated at regular intervals, perhaps each week or each month, much the way ESPN updates all major league baseball statistics every day as soon as each game is completed. Older normative data e.g., (tests administered three or four months earlier) could be eliminated as new data are collected and integrated into the normative sample; that would keep the norms completely up to date from the first day it is published.

Further, the precise positioning of items in an IRT-based CAT permits great accuracy even with short tests (Sinharay 2016). The technology for designing item banks for CAT is sophisticated (van der Linden and Glas 2010; Wilson 2004), including measuring intelligence (van der Linden 2005). And there is precedent for developing thousands of items for the new cognitive tests, using the architecture underlying the automatic item generation program for math tests that can be applied to measure *Gf* (matrices), *Gc* (picture vocabulary), *Gq* (math knowledge), and other abilities as well (T. Matta, Personal communication, 2 and 9 November 2020). 

**Will the Flynn Effect Be a Problem?** By definition, a person’s score on an intelligence test is based on their performance relative to others *at that particular point in time*. The Flynn Effect demonstrates that the norms of every intelligence test are outdated in the U. S. at the rate of 3 global IQ points per decade; they are already a bit out of date the day the test published (typically about a year after the sample is complete). As test norms age, they spuriously inflate a person’s IQ. In clinical practice and in courts of law, IQ points must be subtracted from a person’s obtained IQ based on how old the norms are to remove that spuriousness (Flynn 2020). The proposed continuous norming procedure totally avoids the Flynn Effect.

**Equity.** DIF formulas (Zwick et al. 1995) would be applied during test development phases to eliminate items biased by ethnicity or gender or socioeconomic status. However, to avoid marginalizing any person or any group, I would recommend to the TRADE panel the model used in Europe when standardizing the new tests—neither race nor ethnicity would be included as stratification variables or even recorded. I know personally, by co-authoring a test for French-speaking people, that they take a different approach to stratification variables than we do in America.

The Research Director of our French test, Dr. Louis-Charles Vannier (Personal communication, 12 November 2020) said: “In France, as in Spain, Sweden, Denmark and Norway, we don’t use Race or Ethnicity when we stratify our samples. From a legal standpoint, collecting official census data about race or religious opinion is illegal—incompatible with the first article of the French constitution”. 

**Ease of Administration.** Whereas current clinical tests of intelligence can only be administered by professionals with years of supervised training, usually psychologists, the self-administered CATs can be proctored by nurses, technicians, and others who complete a brief training program. The fact that administration of these tests would not require any physician to be present raises the possibility of AMA approval for inclusion in annual physical exams.

### 6.8. How Might Public Awareness and Approval Be Achieved?

Dr. Dillon (Personal communication, 3 December 2020) cautions, “this testing capability will not be widely utilized unless the general public can be broadly exposed to its potential benefits”. The first task of the TRADE panel would be to raise public awareness of the problem and of the science behind it. Prior to any test development, publicity campaigns would need to teach Americans about the crucial research on the decline in cognitive abilities, such as reasoning and memory, in elderly individuals, and its importance for the National interest. 

Regarding specific steps to take, Dr. Dillon (Personal communication, 15 October 2021) advises:

If a long-term, concerted effort was made to educate the media and professional organizations (ABA, AMA) it could have a huge impact via moral suasion. But it would require a very patient endurance. The moral suasion approach has huge advantages in that it fits smoothly into our political system (we love to point out weaknesses in our government leaders), and could apply to a broad scope of individuals (including federal, state and local government officials and judges). You would have to start with getting a broader group of scientists/academics to verify critical points concerning competency and to formulate a convincing and accessible statement of 2 or 3 crucial concepts for everyday people with a high school education or higher. You recruit a small group of respected scientists/academics to approach sympathetic media outlets and social media personalities to tell that simplified story. You need to keep at this stage until it goes “viral” and becomes the modern equivalent of true. Ultimately, any approach that advocates removal of federal judges is designed for an argument, a large-scale political argument, and would have to face the verdict by a significant population of voters and/or taxpayers.

Brian Hays, JD (Personal communication, 4 January 2021), a specialist in bringing scientific and technology innovations to the healthcare industry, suggests a strategy that targets physicians:

One way to get public approval is to get the American Medical Association to adopt mental testing in annual physical exams that go beyond the current simplistic dementia testing. If the standard of care for general practitioners includes an assessment of reasoning capability, then reimbursement of the cost by Medicare/Medicaid would be a powerful step toward wide acceptance; applying that testing to government officials on an annual basis would not be controversial.

Hays (Personal communication, 15 October 2021), also advises directly involving lawyers to reach a broader audience:

To get the question properly considered, the issue has to move from an academic discussion to become an issue for the legal system to consider. There is actually a well-worn path for this. The topic could first move from the scientific academic community to the legal academic community. The legal scholars can publish in various legal publications, including Law Reviews that are published by virtually every law school. If this paper is introduced to some legal scholars, there is a certainty that the issue would be publicized in the legal press; the actual legal questions are just as interesting as the scientific, social questions. A legal paper could get taken up by the federal bar association or the ABA. Much more to be desired, however, is that a Chief Judge of one of the federal Circuit Courts becomes interested. A Chief Judge has actual power to address the issue, especially if there have been instances of impairment in the Circuit in question.

### 6.9. Why Focus Only on Appointed Federal Judges—Why Not Demand Accountability for Everyone in Power?

In an ideal democratic nation, all candidates for public office, at every level of government from local to national, would be assessed for intellectual function and possible cognitive decline. Seven of the eight proposed tests (all but judiciary expertise, *Gkn*) are ideal assessments for any adult in public service. But for obvious political and historical reasons, the testing of a candidate’s cognitive functions prior to an election or reelection campaign simply will not happen, at least in this millennium. 

Hays (Personal communication, 15 October 2021) offered the following perspective on assessing the competency of anyone in power, whether an elected public official, a physician, a tenured professor, or a judge at the state level:

The question about applicability of the competence standard to other people in power is a fair one. The brutal answer that no one really wants to hear—especially if you are 70 or older—is that every person who has some level of responsibility wider than themselves and their immediate family should be subject to these standards. In many circumstances, there are systems in existence that can be used to question competency. The medical profession (and to a lesser degree the legal profession) has established a ‘standard of care’ to adjudge whether the professional in question is guilty of malpractice.

There is, however, a fundamentally important distinction between all professions and the situation with federal judges. The problem with federal judges is that the appointments are (in effect) lifetime appointments that have very narrow (if any) restrictions on their continuation in their guaranteed position. The check on a President or any Member of Congress—at least in theory—is that they have to stand for re-election. In theory at least (and occasionally in real life) the electorate will not return someone to office if they are demonstrably incompetent. What makes the situation with federal judges particularly acute is that there does not presently exist a realistic check on a lifetime appointment, regardless of the circumstances. As an operating principle for any functioning society, there should be consequences for actions, i.e., accountability. This is especially important in situations where the position holder has power over the life, liberty and property of someone, as a federal judge has.

With judges at the state level, most are elected and, therefore, subject to some level of accountability. In the states where the Governor appoints judges, sometimes based on recommendations from various boards, there is usually a regular review process. Only one state has lifetime appointments for its judges—Rhode Island.

### 6.10. Would the New Tests Have Any Application Besides the Evaluation of Federal Judges? 

Except for the test of judicial expertise, the remaining tests are suitable for any American adult. They might find useful applications for other purposes within the structure of the federal government. For example, Dr. Dillon—one of the first Presidential Appointees enabled by the Civil Service Reform Act of 1978, and a participant in both the Carter and Reagan transition teams—noted:

Another very promising set of public officials where this testing could be exceptionally useful would be in the vetting of so-called Presidential Appointees. These range from Cabinet Secretaries to several thousand lower level officials. People being considered for such positions are routinely vetted and would likely submit to such testing if a new Administration required it as part of the vetting…This might be a unique window of opportunity to introduce the science of intelligence assessments into our political infrastructure (Personal communication, 3 December 2020).

### 6.11. Does this Proposal Reflect Discrimination against the Elderly?

The proposal certainly might appear to be promoting age discrimination, but that is not my interpretation of it. I have focused on the elderly because an array of high-quality empirical studies demonstrates an alarming decline in cognitive abilities with increasing age, especially in problem solving and processing speed. I have also targeted the lifetime appointments of federal judges because of the research-based findings on cognitive decline in the elderly. However, opposition to appointing federal judges is not new. Further, the topic is active right now as I am writing this article. The focus of the commission convened by the Biden administration is whether to add additional seats to the Court. But an additional—less dramatic, but still very important—question up for consideration is whether the Court (and by extension, the rest of the federal judiciary) should be appointed with term limits. According to Abby Phillips (2021), this notion has support among the bipartisan commission. Congress could conceivably consider a law or Constitutional change to limit the terms of federal justices. 

Regarding age discrimination, the paper recommends testing *all* sitting and future federal judges regardless of their age; it also suggests applying the same pass-fail criteria for all judges. Further, the accumulated data indicate that notable decreases in cognitive test scores begin in early middle age. Figure 1 makes it apparent that nonverbal abilities start to decrease steadily and substantially at about age 40. Figure 2 highlights, more specifically, the rapid declines in reasoning and speed that begin well before old age.

The data from all aging-IQ studies are for groups. Within each group, there are broad individual differences on all cognitive abilities across the entire age range. Everyone knows brilliant men and women who are 70 or 80 or 90, including public figures such as federal judges. Also, Hertzog (2020) points out that there are important ways that people can have some say in how well they maintain their cognitive abilities: “Over the past two decades, compelling evidence has emerged that aerobic exercise in middle age and old age promotes enhanced cognitive function in older adults” (p. 196). 

But regardless of political correctness or politics in general, there needs to be a check on the cognitive functioning and judicial expertise of any person who makes high stakes decisions, especially judges who are, in effect, appointed for life.

### 6.12. Is the Proposal to Evaluate the Competency of Federal Judges Legal?

To address this key practical issue, I consulted Thomas Dillon, PhD, and Brian Hays, JD, both of whom possess relevant backgrounds and pertinent experience.

Thomas Dillon (Personal communication, 8 October 2021):

Your proposal would be legal if the Congress passed a law to establish such a procedure for removal of federal judges. However, such a law would need to be judged constitutional, and there is no guarantee of that happening. The best chance would be to pass a law that would use your process as a legitimate basis for impeachment. The Constitution’s only requirement for term of office for all federal judges—Supreme, Circuit, and District—is good behavior. You could claim that your tests could prove that judges no longer had the capacity for good behavior; but even that law would face a political battle regarding its constitutionality and ultimate implementation. So, yes, your proposed process has a pathway to be declared legal and constitutional, but not an easy one”.

Regarding impeachment, Dr. Dillon added, “And, yes, the complicated political process of impeachment would be required. Although, it is important to note that all 8 impeachment convictions were of federal judges” (Personal communication, 9 October 2021).

Brian Hays (Personal communication, 8 October 2021) noted that “passing a law, or a constitutional amendment, are two difficult ways to implement a TRADE program. However, there are other less dramatic and vastly more practical ways to accomplish the goal of pushing toward solid mental capabilities in the federal judiciary”.

Hays explained:

From the point of view of the American federal legal system, the issue is not whether some judges are impaired; the issue is what to do about it. The legal issues are daunting, but there are options that can sidestep the most formidable ones. For example, your concept of a TRADE process would fit in very well in the existing Circuit Judicial Council structure. The Chief Judge has a very broad set of actions that he or she could take to deal with a situation when the TRADE panel determines that a judge is impaired. If the judge in question refuses to resign or even go on to Senior status after gentle prodding, the Chief Judge of a circuit and/or the Judicial Council could effectively remove a federal judge (District or Circuit Court, not Supreme Court) without getting anywhere close to a Constitutional issue—provided the judge in question is allowed to retain their position and salary, but is assigned no cases to adjudicate.

Note that federal judiciary funding is provided by Congress; it would be appropriate for Congress to authorize TRADE funding as part of the annual appropriations process. Note, also, that to trigger the ‘good behavior’ clause of the Constitution in the situation of Supreme Court Justices, the mental impairment would have to be extreme (Personal communication, 9 October 2021).

## 7. Final Thoughts

CHC is but one theory of intelligence and cognitive tests are only one way of measuring a person’s intelligence and competence—and an incomplete way at that. As alluded to previously, Robert Sternberg’s (2020) augmented theory of successful intelligence goes beyond psychometric tests and includes creativity, practical intelligence, the ability to capitalize on strengths to compensate for weaknesses, and the notion of wisdom, all for the purpose of serving the common good via ethical values; his theory of adaptive intelligence (Sternberg 2019) focuses on adaptation to the environment and finding solutions to real-world problems like pollution, global warming, and poverty. Also, Daniel Kahneman’s (2011) life’s work, theories, and integration of research focus on the psychology of judgment, decision-making, overcoming first-level perceptions and biases, self-regulation, and inhibition. In his latest book, Kahneman et al. (2021) argue that in any process that includes judgment, noise is inherent. They specifically discuss the variability in determining sentences imposed by federal judges for convicted criminals.

Sternberg’s and Kahneman’s theories and research address political competence and practical solutions to real-world problems, and these attributes of intelligence are only partially measured by standardized tests of cognitive ability. As discussed earlier, the TRADE panel needs to affirm that the abilities proposed for the new set of tests are relevant to a judge’s competence, and they must develop a reliable and valid criterion measure of judicial expertise. Also, they should carefully consider developing additional tests that are pertinent to a judge’s job performance, especially the kind that measure the real-life abilities and skills studied by theorists and researchers such as Sternberg and Kahneman.

Though necessarily incomplete as measures of all of intelligence, the psychometric CHC theory and the research it has spawned provide the scientific foundation for moving forward with the identification of those federal judges who should undoubtedly be urged to resign rather than serve for the rest of their lives. There is strong empirical support for predictable, and differential, changes in key cognitive abilities between young adulthood and old age, a body of research that I believe runs contrary to lifetime appointments for Supreme Court justices and other federal judges. We also have access to advanced technology for the development of reliable and valid computerized tests, administered remotely if necessary (Wright and Raiford 2021; Wright 2020), to assess these abilities. It is now time for a bipartisan movement across the U. S. to put psychological science into action. 

## Data Availability

Data are owned by Pearson. Permission was obtained to reprint the figures showing the data, by both Pearson and Wiley. But the dataset is not available to the public.
